# Reconciling fish and farms: Methods for managing California rice fields as salmon habitat

**DOI:** 10.1371/journal.pone.0237686

**Published:** 2021-02-24

**Authors:** Eric J. Holmes, Parsa Saffarinia, Andrew L. Rypel, Miranda N. Bell-Tilcock, Jacob V. Katz, Carson A. Jeffres

**Affiliations:** 1 Center for Watershed Sciences, University of California Davis, Davis, California, United States of America; 2 Department of Wildlife, Fish & Conservation Biology, University of California Davis, Davis, California, United States of America; 3 California Trout Inc., San Francisco, California, United States of America; Tanzania Fisheries Research Institute, UNITED REPUBLIC OF TANZANIA

## Abstract

Rearing habitat for juvenile Chinook Salmon (*Oncorhynchus tshawytscha*) in California, the southernmost portion of their range, has drastically declined throughout the past century. Recently, through cooperative agreements with diverse stakeholders, winter-flooded agricultural rice fields in California’s Central Valley have emerged as ecologically functioning floodplain rearing habitat for juvenile Chinook Salmon. From 2013 to 2016, we conducted a series of experiments examining methods to enhance habitat benefits for fall-run Chinook Salmon reared on winter-flooded rice fields in the Yolo Bypass, a modified floodplain managed for flood control, agriculture, and wildlife habitat in the Sacramento River Valley of California. Investigations included studying the effect of 1) post-harvest field substrate; 2) depth refugia; 3) duration of field drainage; and 4) duration of rearing occupancy on in-situ diet, growth and survival of juvenile salmon. Post-harvest substrate treatment had only a small effect on the lower trophic food web and an insignificant effect on growth rates or survival of rearing hatchery-origin, fall-run Chinook Salmon. Similarly, depth refugia, created by trenches dug to various depths, also had an insignificant effect on survival. Rapid field drainage yielded significantly higher survival compared to drainage methods drawn out over longer periods. A mortality of approximately one third was observed in the first week after fish were released in the floodplain. This initial mortality event was followed by high, stable survival rates for the remainder of the 6-week duration of floodplain rearing study. Across years, in-field survival ranged 7.4–61.6% and increased over the course of the experiments. Despite coinciding with the most extreme drought in California’s recorded history, which elevated water temperatures and reduced the regional extent of adjacent flooded habitats which concentrated avian predators, the adaptive research framework enabled incremental improvements in design to increase survival. Zooplankton (fish food) in the winter-flooded rice fields were 53-150x more abundant than those sampled concurrently in the adjacent Sacramento River channel. Correspondingly, observed somatic growth rates of juvenile hatchery-sourced fall-run Chinook Salmon stocked in rice fields were two to five times greater than concurrently and previously observed growth rates in the adjacent Sacramento River. The abundance of food resources and exceptionally high growth rates observed during these experiments illustrate the potential benefits of using existing agricultural infrastructure to approximate the floodplain wetland physical conditions and hydrologic patterns (shallow, long-duration inundation of cool floodplain habitats in mid-winter) under which Chinook Salmon evolved and to which they are adapted.

## Introduction

The development of multi-benefit land use practices that reconcile the needs of human societies with ecosystem function are critically important to biodiversity conservation given human population growth and the concurrent expansion of terrestrial land surface dedicated to agriculture [1, 2]. Accordingly, reconciliation ecology, which is the practice of encouraging biodiversity in the midst of human dominated ecosystems by specifically managing the landscape for the benefit of fish and wildlife has become an increasingly important component of global conservation efforts [3, 4]. This is especially true in freshwater habitats which constitute less than 1% of Earth’s land surface yet support freshwater fish species that make up approximately one third of all known vertebrates [5] and where loss of biodiversity appears to be more rapid than in any other habitat type [6, 7]. Even among imperiled freshwater habitats, rivers and their associated floodplains stand out as among the most altered ecosystems in the world [8, 9]. They are also among the most desirable and agriculturally productive landscapes globally and therefore ideal locations for case-studies on innovative reconciliation ecology-inspired, multi-benefit land use innovations [10]. Furthermore, these lands are managed to perform economically valuable functions of human food production and flood risk mitigation while simultaneously providing critical ecosystem benefits such as nutrient cycling, aquifer recharge, habitat creation, and conservation of biodiversity in heavily altered landscapes [11, 12].

Managing agricultural floodplain habitats in ways that approximate natural riverine processes re-exposes native species to physical habitat conditions similar to those to which they are adapted and may therefore enhance fitness and survival [13, 14]. To date, most North American work to reconcile working agricultural floodplain farmlands with the needs of wildlife has focused on waterfowl conservation [15, 16]. However, in Asia fish have been reared in rice fields for thousands of years, providing a valuable protein resource, natural fertilizer for agricultural fields, and refugia/food for native fishes [17, 18]. This paper explores means by which fish conservation can be integrated into the management of actively farmed rice fields (already being managed to benefit migratory bird populations) on the agricultural floodplains of the Sacramento Valley, California.

Chinook Salmon (*Oncorhynchus tshawytscha*) are in steep decline throughout California [19]. A conservative pre-European establishment fish population estimate in the Central Valley was 2 million annual adults returning to spawn, which sustained a sizable commercial ocean fishery [20]. Prior to the mid-1800s, California’s Central Valley was estimated to contain more than 4 million acres of seasonal floodplain and tidal wetlands which provided abundant food resources for rearing juvenile Chinook Salmon [21]. Of the historic wetland habitats in California, approximately 95% of floodplain habitat has been disconnected from rivers by levees and channelization, drastically reducing quality rearing conditions for out-migrating salmon [22, 23]. Though most of the historical alluvial floodplain in California is now inaccessible to salmon, some productive seasonal wetlands persist, presenting opportunities for conservation. In particular, winter-flooded rice fields within the Sacramento Valley flood protection bypasses–floodways which route floods away from cities and which are designed to drain flood waters rapidly in order to accommodate agricultural production–hydrologically connect to the river and can be managed to promote environmental conditions that resemble natural off-channel habitat [24–27].

Use of existing berms and water control structures used in rice propagation to prolong the duration of floodplain inundation on these managed floodplain wetlands during the winter and early spring seasons approximates the long-duration inundation of floodplains that typically occurred on Central Valley floodplains prior to the widespread wetland reclamation and levee construction in the 19th and 20th centuries. Inundation duration of several weeks (typically 3–6 weeks) facilitates the development of highly productive invertebrate food webs and improved foraging opportunities for fish [28]. Chinook Salmon reared in floodplain and off-channel habitats experience more rapid growth rates compared to those rearing in adjacent leveed river channels rivers due to more abundant invertebrate prey [24, 29]. For anadromous salmonid species such as Chinook Salmon improved growth during the freshwater juvenile stage is correlated with larger size at ocean entry and increased survivorship to adulthood [30–33]. While the potential benefits to juvenile Chinook Salmon rearing on flooded bypasses is well established [24, 26, 27, 29], there is little published research testing methodologies for establishing the optimal physical and biological conditions to achieve maximal benefit on these managed floodplains.

Such is the primary goal of this study: to compare potential management practices intended to enhance the habitat benefits to juvenile Chinook Salmon of winter-inundated, post-harvest rice fields on the Yolo Bypass floodplain of the Sacramento Valley of California. This paper reports results from work conducted on a 7.3-hectare agricultural floodplain laboratory over four consecutive years beginning in 2013 and ending in 2016. Studies were built on an adaptive framework in which each year’s results are used to refine experimental approaches in subsequent field seasons. Listed sequentially, annual investigations included studying the effects of 1) post-harvest field substrate; 2) depth refugia; 3) duration of field drainage; and 4) duration of rearing occupancy on in-situ diet, growth and survival of juvenile salmon. It is our hope that the data produced by these controlled, field-scale experiments will inform farm, water, and flood resource managers as they continue to develop multi-benefit land use practices designed to improve habitat quality for salmon and other native fishes of conservation concern provided California’s system of water supply and flood protection infrastructure.

## Methods

### Study animal

The fish used in the experiments detailed below were hatchery-raised juvenile fall-run Chinook Salmon sourced from the California Department of Fish and Wildlife Feather River State Fish Hatchery. Following transport from the hatchery, fish were acclimated to the physical water conditions by incrementally mixing rice field water with the transport water before release into the experimental plots. Animal subjects were handled in strict accordance with the specific guidelines for these experiments issued from the University of California, Davis Institutional Animal Care and Use Committee (Protocol # 18883) and the California Department of Fish and Wildlife (Scientific Collection Permit: 8539).

### Study area

**Fig 1:**
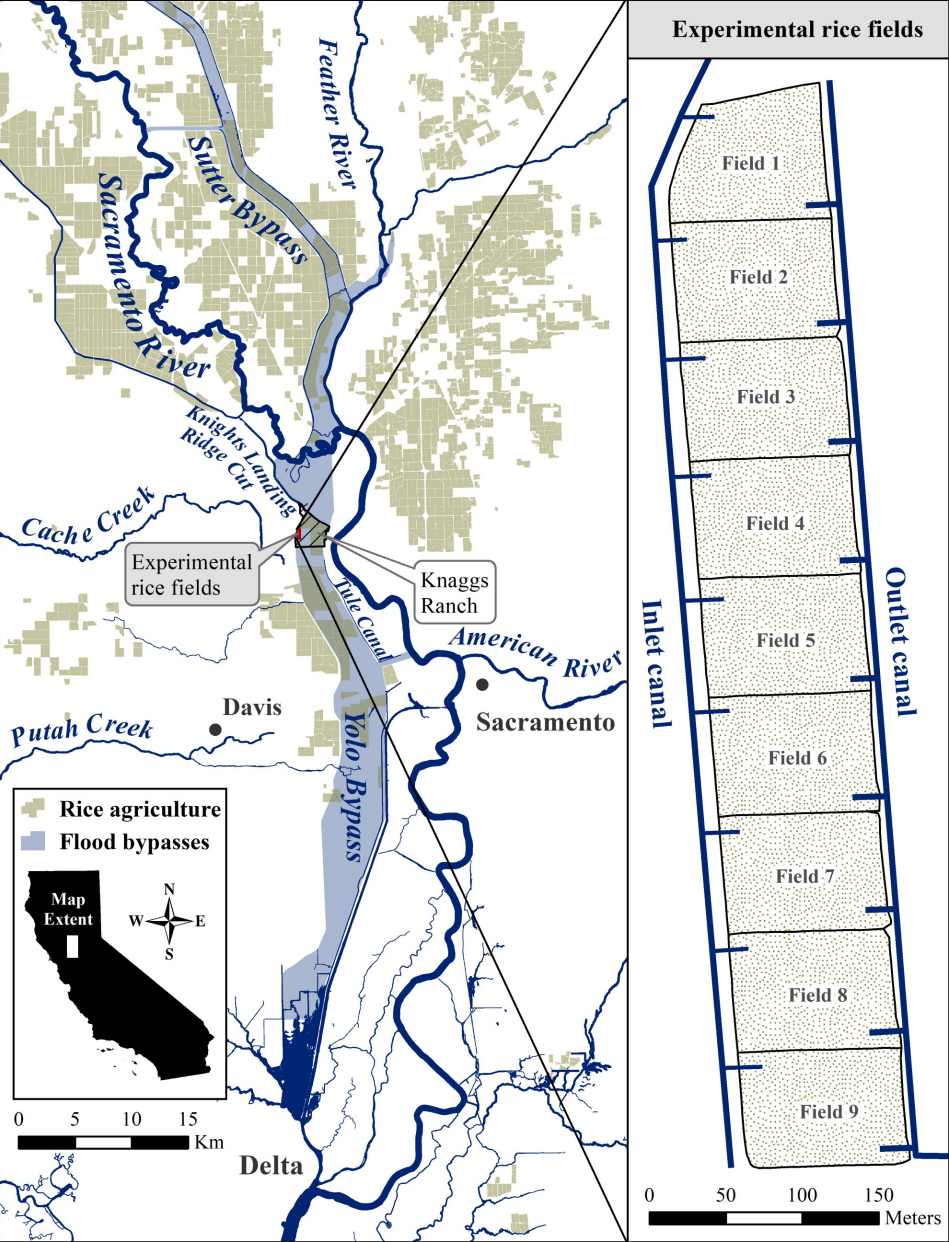
Map of the study area. Schematic of the nine experimental rice fields (right) located at Knaggs Ranch in geographic context of the major water features and flood bypasses of the lower Sacramento Valley (left). Rice agriculture spatial coverage was from the 2016 California statewide agricultural land use dataset provided by the California Department of Water Resources.

Experiments took place in the Yolo Bypass, a 24,000-ha flood bypass along the Sacramento River in California, USA. Nine 0.81-ha replicated fields were constructed on Knaggs Ranch—a farm predominantly producing rice (Fig 1). An inlet canal routing water from the Knights Landing Ridge Cut canal independently fed each of the nine fields, and all fields drained into an outlet canal. The outlet canal ultimately emptied into the Tule Canal, which runs north to south along the east side of the bypass. Each field had rice boxes (structure using stacked boards to control water elevation and flow) on the inlet and outlet of each field. Water depths, as measured in the middle of the fields, were maintained between 0.3 m to 0.5 m for all years. Inlet structures were fitted with 3-mm mesh screens to permit water inflow and prevent egress of stocked salmon. Outlet structures were fitted with 3-mm mesh screens in the 2013 and 2016 experiments. However, in 2014 and 2015, outlet structures were left open with a 5-cm diameter hole drilled in the middle of a 3.8-cm × 14-cm board and placed near the top of the water level in the rice box to investigate volitional outmigration patterns of the stocked salmon. Each outlet structure was fitted with a live car trap placed in the outlet canal, which allowed for collection of all exiting fish. In 2014 and 2015, live cars were checked daily for the duration of the experiments to enumerate the number of emigrating salmon. In past experiments we observed a tendency for a portion of hatchery fish to “scatter” upon initial release into floodplain fields. This behavior reliably abated after several days as fish acclimated to new conditions. For this reason, downstream exiting fish were restocked back to the inlet side of the fields for the first week of 2014. In 2015, fish were similarly restocked for two weeks.

### Experiments

#### Substrate type– 2013

After harvest, rice farmers typically treat the residual rice straw remaining in the fields using one of several methods; thus an important question was whether differences in treatment of rice straw created different outcomes for rearing fish. Nine fields were randomly assigned to one of three post-harvest substrate treatments: rice stubble, disced, or fallow. The rice stubble substrate treatment consisted of standing stalks (heights ranging from 0.23–0.35m) that remained after rice plants were cut for harvest using a rice harvesting combine tractor. The disced treatment consisted of plowing rice straw into the soil, a practice farmers use to promote stubble decomposition. The fallow habitat had not been planted with rice during the previous growing season but instead consisted of weedy herbaceous vegetation that voluntarily colonized the fields during the growing season and was left standing during the experiment. More details on the 2013 experimental design can be found in publications by our colleagues [24, 34].

#### Depth refugia– 2014

Avian predation on fish in aquaculture fields is a well-known problem [35–37]. Avian predation has the potential to be a significant source of fish mortality in winter-flooded rice fields as California’s Central Valley is positioned directly within the winter habitat of diverse bird populations in the Pacific flyway [38, 39]. We evaluated whether trenching could provide depth refuge as a potential method for reducing avian predation on fish in winter-flooded rice fields. In 2014, nine fields all with a disced substrate, were randomly assigned to one of three treatments: three fields were assigned no perimeter trench, three were assigned a 0.5 m deep perimeter trench, and three were assigned a 1.0 m deep perimeter trench. All trenches were constructed on the north and east sides of the fields running continuously from the inlet structure in the northwest corner to the drain structure in the southeast corner. All trenches were approximately 1.0 m wide with the outermost edges of the trench spaced approximately 1.0m from the exterior levee surrounding the field. We created this spacing specifically so depth refuges were outside the striking distance of wading birds such as herons and egrets which frequent the shallow water of the perimeter levees. Survival data for three fields was excluded from the analysis due to loss of containment on the inlet side of three fields (fields 3, 4, and 7, one from each treatment) during the last week of the experiment allowing fish to escape upstream into the inlet canal. Ancillary effects of the trench treatments on field drainage efficiency and volitional migration of fish were also investigated.

#### Drainage practices– 2015

Floodplain hydrology provides important cues for movement and egress of floodplain species [40, 41]. We wanted to know if we could create artificial hydrologic cues to trigger fish out-migration (volitional egress) from fields. To investigate drainage practice effects on fish survival, the nine fields were randomly assigned one of three draining treatments: 1) fast drain, where inlet water was cut off and outlet boards were removed rapidly, resulting in the water draining off the fields in a single day; 2) slow drain with inflow, where water levels were lowered by 5 cm per day at the outlet while inflow was maintained through a mesh screen; and 3) slow drain without inflow, where water levels were lowered 5 cm per day at the outlet and inflow was cut off by boarding up the inlet structure. The drainage duration for both slow drain procedures lasted for 10 days with daily outmigration of salmon measured in the outlet traps. All nine experimental fields had a rice stubble substrate following the rice harvest in fall 2014, and a 0.5 m deep perimeter trench was constructed in all fields connecting the inlet and outlet structures running along the north and east sides of the fields. The trenches were approximately 1.0 m wide and spaced 1.0 m infield from perimeter levees.

#### Survival through time– 2016

To examine in-field survivorship of juvenile salmon through time, fish were stocked in six of the nine flooded experimental fields. During each of following six weeks, one randomly selected field was drained using the fast drain procedure, detailed in the 2015 experiment. All fields had fallow substrate as described in the 2013 experiment and 0.5 m deep trenches as described in the 2015 experiment. An impending bypass flood event near the end of the study forced the drainage of the last field 4 days earlier than scheduled.

#### In-field water quality

Across all years and fields, we recorded continuous water temperatures in 15-min intervals using HOBO U22 temperature loggers (Onset Computer Corporation, Bourne, Massachusetts, USA) anchored in a fixed vertical position on a metal t-post approximately 10 cm above the substrate in the middle of each field as well as trench substrate for a representative set of treatments when applicable. Localized temperature refugia in the trenches was evaluated in its capacity to create thermal buffering by comparing daily maximum water temperatures in the bottom of a trench to those in the middle of the field. Analysis of other physical water quality parameters, nutrient loading, and primary productivity in these experimental rice fields can be found in publications by our colleagues [24, 29].

#### Zooplankton abundance

Throughout all years, a randomly stratified subset of three fields was sampled for zooplankton weekly except in 2013 where all nine fields were sampled weekly. A 30-cm diameter 150-μm mesh zooplankton net (with the exception of the 2016 experiment, which used a 15-cm diameter 150-μm mesh net) was thrown 5 m and retrieved through the water column four times, once in each cardinal direction. In 2013, benthic macroinvertebrates were sampled separately using benthic sweeps, but due to high sedimentation, high spatial and temporal sample replication, and low overall contribution to the invertebrate community, the additional processing was deemed unnecessary in subsequent years. Furthermore, the zooplankton tow method is effective for assessing pelagic zooplankton and macroinvertebrate community assemblages while improving sample processing efficiency since it avoids the heavy sedimentation associated with benthic sweeps on wetland substrates [42]. Additionally, we also relied on the stomach contents of in-field salmon to better inform the assemblage of macroinvertebrates present in the floodplain food web and their contribution to the diet of in-field salmon (methods in next section). Sampling location in each test plot was determined randomly via a selection of random x and y distances from a random number table. All samples were preserved in a solution of 95% ethanol. Organisms were identified with the aid of a dissecting microscope at four times magnification to the lowest taxonomic level possible using several widely recognized keys [43–45]. Abundance estimates were calculated from homogenized subsamples of known volume and extrapolated to the volume sampled during the initial net throws.

#### Salmon stomach contents

A random sub-sample of in-field salmon captured during weekly sampling with 4.8-mm mesh seine (2013–2015) and sequential field draining (2016) were sacrificed, transported on ice, and stored in a freezer at -22°C. A total of 532 salmon stomachs (2013: n = 268, 2014: n = 144, 2015: n = 90, 2016: n = 30) were dissected using a dissecting microscope at four times magnification. Prey items were enumerated, but due to variable decomposition, prey item identification in the stomachs was limited to taxonomic order.

#### Overall salmon survival and growth

Estimates of initially stocked salmon in each field were calculated by establishing a fish per kilogram ratio and multiplying by the total weight applied to each field, except in 2016 where the overall number of stocked fish was sufficiently low to count individually. Stocking density was calculated by dividing the estimate of initially stocked salmon by the field area (Table 1). Fish lethally sampled for stomach content analysis during weekly sampling were subtracted from the initial stocking estimate. Total salmon survival in each field was cumulatively enumerated in the outlet live car traps except during the restocking phase of 2014 and 2015 when volitionally emigrating fish were restocked to the inlet side of the fields. During field drainage, seines were used to collect stranded fish out of standing water and these fish were added to the cumulative survival count from the outlet live cars with the recovery method recorded. Survival in 2015 was calculated from only the fast drain treatment fields since the drawn out drainage methods were not comparable to drainage methods in other years.

**Table 1:** Summary of salmon stocking dates, experiment durations, stocking densities (fish m^-2^), mean initial fork length (mm), mean initial wet weight (g), and mean final fork length (mm) and mean final wet weight (g).

Year	Stocking date	Experiment duration (days)	Stocking density (fish m^-2^)	Mean initial FL (mm)	Mean initial wet weight (g)	Mean final FL (mm)	Mean final wet weight (g)
**2013**	Feb 19	37–41	0.57	53	1.53	87	7.96
**2014**	Feb 4	36–45	0.59	43	1.01	80	5.94
**2015**	Feb 5	22–32	0.49	50	1.37	67	3.88
**2016**	Feb 1	7–38	0.12	40	0.68	87	8.71

Prior to stocking in each year, mean initial fork length and wet weight were calculated from a random sample of 30 live fish measured to the nearest millimeter and weighed to the nearest hundredth of a gram with an Ohaus Scout Pro SP202 scale (Table 1). For 2013–2015, we conducted weekly in-field fish sampling with a 4.8 mm mesh seine to capture a target of 30 fish per treatment, with the fork length and wet weight measured. In 2016, fish size data were collected from a random sample of 30 fish in out-migrant traps as individual fields were drained weekly.

### Statistical analysis

Percent survival for each field was calculated as the total number of recovered fish divided by number of initially stocked fish, times 100. Analysis of covariance (ANCOVA) was used to test for interaction effects between field substrate treatment and time which would indicate treatment effects on salmon growth rates. In this model, fork length was the dependent variable with field substrate, day of the experiment and an interaction term as the independent variables. When the assumptions of normality and homogeneity of variance were satisfied, as tested by the Shapiro-Wilk and Levene tests respectively, a one-way analysis of variance (ANOVA) was used to test for significant differences in survival due to field drainage treatments. A post hoc Tukey honestly significant differences (HSD) test was used to test all pairwise comparisons of field drainage practices. When the assumptions of normality and/or homogeneity of variance were not satisfied, non-parametric Kruskal-Wallis analysis was used to test for significant differences in survival and daily volitional outmigration due to field trench depth treatments and to test for differences in overall mean total zooplankton densities between years and substrate types. A post hoc Dunn’s test was used to test all pairwise comparisons of daily volitional outmigration due to field trench depth treatments. Linear regression was used to estimate apparent growth rates and to examine the relationship between salmon survival (dependent variable) and day of the experiment (independent variable). Linear regression was also used to evaluate the degree of within-field thermal refugia via the relationship between daily maximum water temperature differences in the trenches (dependent variable) and daily maximum water temperature in the middle of the field (independent variable). Statistical significance was declared at an α = 0.05 level. All analyses were conducted in R v3.6.1 [46].

## Results

### Substrate type– 2013

Apparent fork length growth rate for juvenile salmon did not differ significantly between treatments (ANCOVA, F = 2.16, df = 2, P = 0.11). The slopes from individual linear regressions of fork length predicted by day for each treatment resulted in estimated apparent growth rates of 1.01 mm d^-1^ for the stubble treatment, 0.99 mm d^-1^ for the disced treatment, and 0.95 mm d^-1^ for the fallow treatment.

As previously published [24], found no statistical difference between total abundance of zooplankton between treatments, but did find high overall abundance and a trend of increasing zooplankton over experiment duration. Across all samples, cladocera were the most abundant group of zooplankton, making up over 50% of the total zooplankton assemblage.

Cladoceran zooplankton was the most common prey item found in juvenile salmon stomach contents as this taxon comprised on average 94.0% ± 1.0% SE of the diet composition across all treatments. Chironomid midges (diptera) were the second most common prey item and comprised an average of 4.8% ± 1.0% SE of the diets. Diet composition was slightly more diverse in the fallow treatment with an average of 87.3% ± 2.6% SE percent of prey items composed of cladocerans compared with an average of 97.4% ± 1.2% SE in the disced treatment and 97.3% ± 1.0% SE in the stubble treatments. A chironomid midge hatch in the southernmost field (field 9) was responsible for the increased prey diversity resulting in diets composed of an average of 69% cladocera and 30% diptera. The other two fallow replicates had an average diet composition of 96% cladocera.

### Depth refugia– 2014

Depth treatments did not have a significant effect on survival (Kruskal-Wallis, χ^2^ = 0.86, df = 2, P = 0.65). Depth treatments, did however have a significant effect on daily volitional emigration of fish before draining (Kruskal-Wallis, χ^2^ = 14.70, df = 2, P < 0.001). A post-hoc Dunn’s test revealed that the two trenched treatments had significantly more daily volitional outmigration compared to the no trench treatment (Dunn test, 0.5m trench–no trench: P < 0.001, 1m trench–no trench: P = 0.003), but that the two trench treatments did not significantly differ from each other (0.5m trench– 1m trench: P = 0.64). The average cumulative volitional outmigration before field drainage in the two trenched treatments was 15.4% ± 5.3% SE compared to the trenchless treatment, which had 3.3% ± 1.2% SE, indicating the trenches may have functioned as a migratory pathway aiding in volitional outmigration prior to field drainage. A relatively high rate of initial volitional emigration was seen in the first week (1.5%) across all fields, followed by a much lower rate of emigration in the second week (0.2%), and steadily increasing emigration in weeks three through five (0.5%, 1.6%, 5.6% respectively). Manual fish recovery with a seine at the end of field drainage in the trenchless fields ranged between 5 to 20% of the total surviving fish compared to less than 0.5% of survivors from the trenched fields which indicated a more efficient drainage procedure in trenched fields. Again there appeared to be functional equivalence between the 0.5m and 1.0m trench treatments.

### Drainage practices– 2015

**Fig 2:**
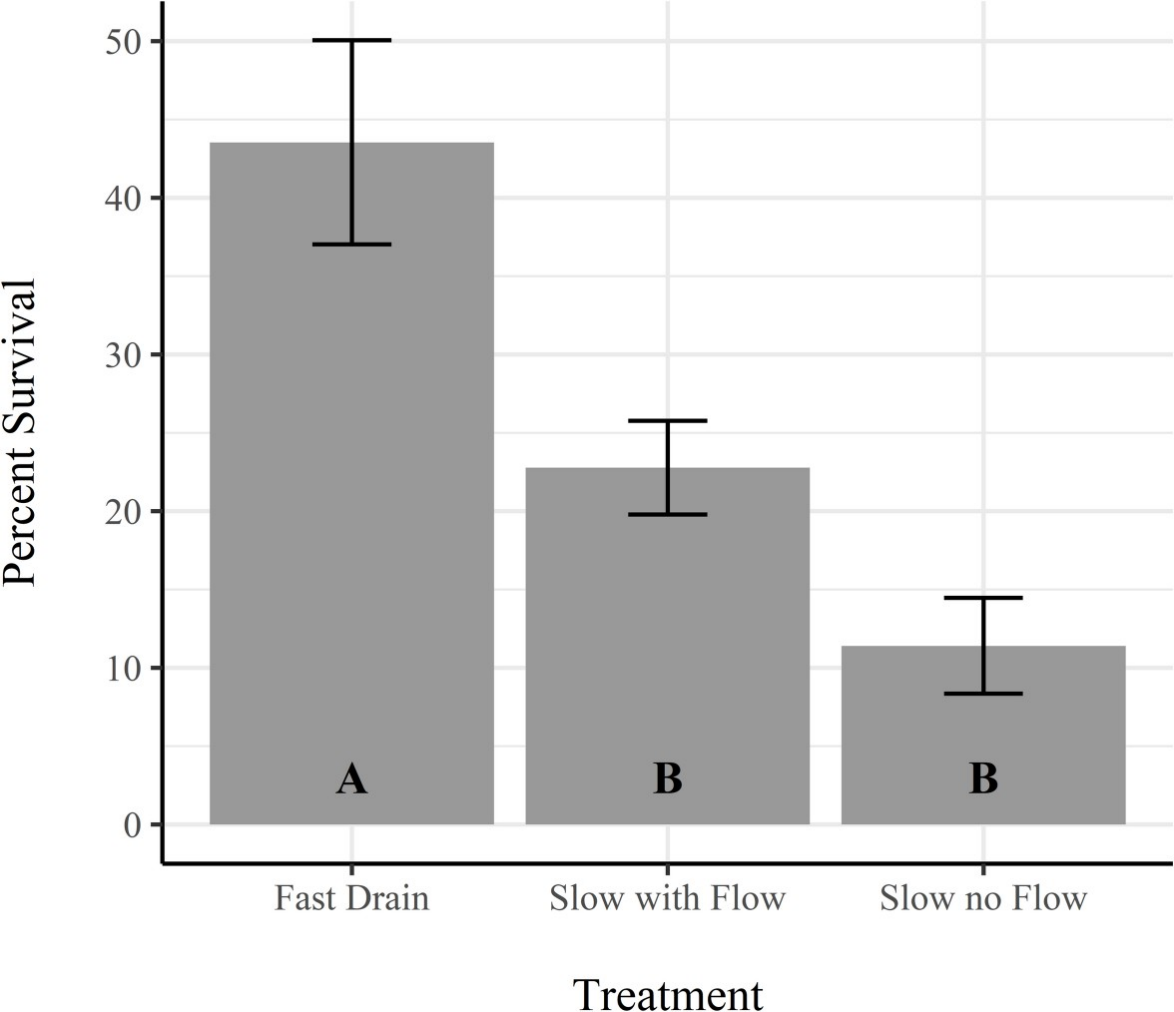
Survival response to drainage treatment. Bars represent mean percent survival for each drainage treatment from the 2015 experiment. The error bars denote standard errors. Letters on the bars denote significantly distinct treatment groups.

Average salmon survival in each of the three treatments, fast drain, slow drain with flow, and slow drain without inflow, was 43.5% ± 6.5% SE, 22.8% ± 3.0% SE, and 11.4% ± 3.1% SE respectively (Fig 2). The differences between field drainage treatments were significant (ANOVA, F = 13.15, df = 2, P = 0.01). A post hoc Tukey HSD analysis revealed that pairwise comparisons of survival in the fast drain treatment were significantly higher than either slow drain treatment (P = 0.04 and P = 0.01 for slow with flow and slow without flow treatments respectively). Survival between the two slow drain treatments were not significantly different from each other (P = 0.25). Volitional outmigration patterns were similar to 2014 with relatively high initial emigration in the first week (2.7%), low emigration in the second week (0.2%), and high emigration in week three (3.8%).

### Survival through time– 2016

**Fig 3:**
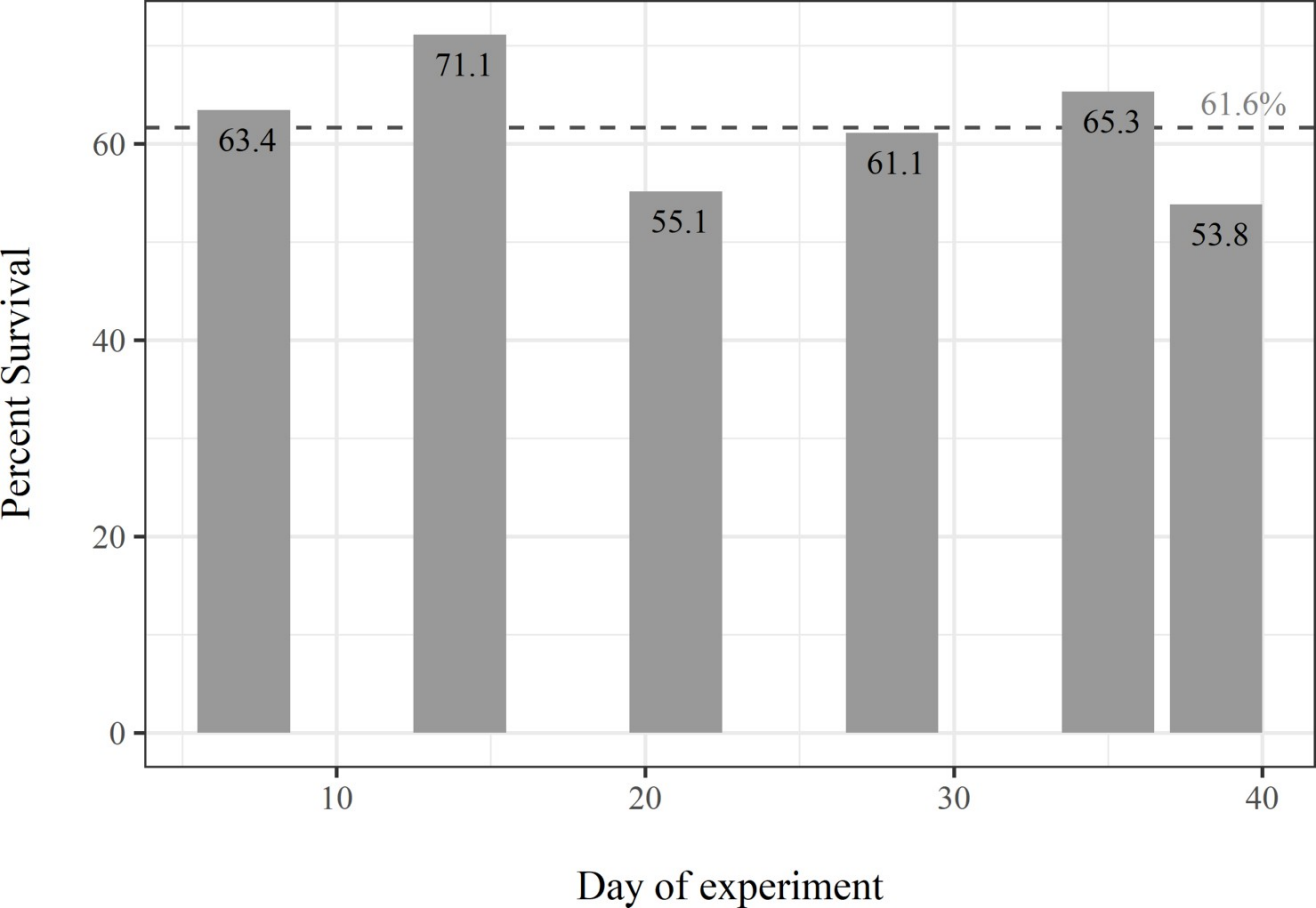
Survival over time. Bars represent percent survival of stocked salmon over time in sequentially drained fields from the 2016 experiment. The dotted line represents the mean percent survival from all fields.

Across all draining durations, in-field survival of juvenile salmon averaged 61.6% ± 6.5 SD, with the final field survival being 8.0% lower than the first field drained 31 days earlier (Fig 3). The slope of a linear survival regression model predicted by day (range: 7–38 days) was -0.24% per day with an intercept of 67.4%. Due to low sample size (n = 6) and inherent variability in overall survival, the linear survival model had a low adjusted R^2^ and non-significant P-value (Regression, F = 1.05, df = 4, P = 0.36, R^2^ = 0.01), however, the model coefficients indicate a relatively low attrition rate (slope) after a substantial initial loss (intercept) of approximately one third during the first week of the experiment.

### In-field water quality

Continuously logged water temperatures at the center of the fields during experiments from all years ranged between 5.5°C and 23.5°C. Water temperatures exceeding 21°C, which can negatively affect growth potential and predator avoidance [47], were experienced 1.6% of the time. Trenches generated thermal refugia in the bottoms of the trenches with lower daily maximum water temperatures compared to the middle of the fields by an average of 1.0 ± 0.22 SE°C and 1.8 ± 0.25 SE°C for the 0.5 m and 1.0 m trenches, respectively. There was a significant correlation between maximum daily water temperature in the middle of the fields and the difference between the maximum daily water temperature in the middle of the field and bottom of the 0.5 m and 1.0 m deep trenches (0.5 m trench: regression, F = 10.4, df = 1,23, P = 0.004; 1.0m trench: regression, F = 43.89, df = 1,31, P < 0.001). Over the observed range of daily maximum water temperatures in 2014 and 2015 of 11.4 to 21.1°C, the regression slopes showed 0.26°C and 0.60°C temperature reductions in the trenches per degree increase in the middle of the field for the 0.5 m and the 1.0 m trenches respectively.

### Overall zooplankton

Measured zooplankton densities during the experiment ranged from a low of 14,961 organisms m^-3^ on Feb 1st, 2016 in field 2 (fallow substrate) to a high of 231,966 organisms m^-3^ on Mar 20th, 2013 in field 6 (disced substrate). Overall mean zooplankton density was consistent each year and ranged from a low of 75,045 organisms m^-3^ in 2016 to a high of 107,039 organisms m^-3^ in 2015 with no significant differences detected between years (Kruskal-Wallis, χ^2^ = 5.44, df = 3, P = 0.14, Table 2). Overall mean zooplankton densities for substrate across all years were 82,191 ± 8,697 SE, 81,283 ± 5,804 SE, and 93,585 ± 9,703 SE organisms m^-3^ for the disced, fallow, and stubble treatments respectively, with no significant differences detected (Kruskal-Wallis, χ^2^ = 1.57, df = 2, P = 0.45). These results indicated consistently high abundance of zooplankton in the fields in all years and across all substrate types.

**Table 2:** Mean in-field zooplankton density m^-3^, mean percent cladocera from in-field zooplankton samples, and mean percent cladocera in salmon diets.

Year	Mean total zooplankton density (m^-3^)	Mean percent cladocera in fields	Mean percent cladocera in diets (%)
**2013**	81,417 ± 7,189 SE	56.0%	94.0%
**2014**	96,768 ± 11,066 SE	52.9%	95.0%
**2015**	107,039 ± 14,351 SE	25.2%	92.5%
**2016**	75,045 ± 6,605 SE	16.4%	91.2%

### Overall salmon stomach contents

**Fig 4:**
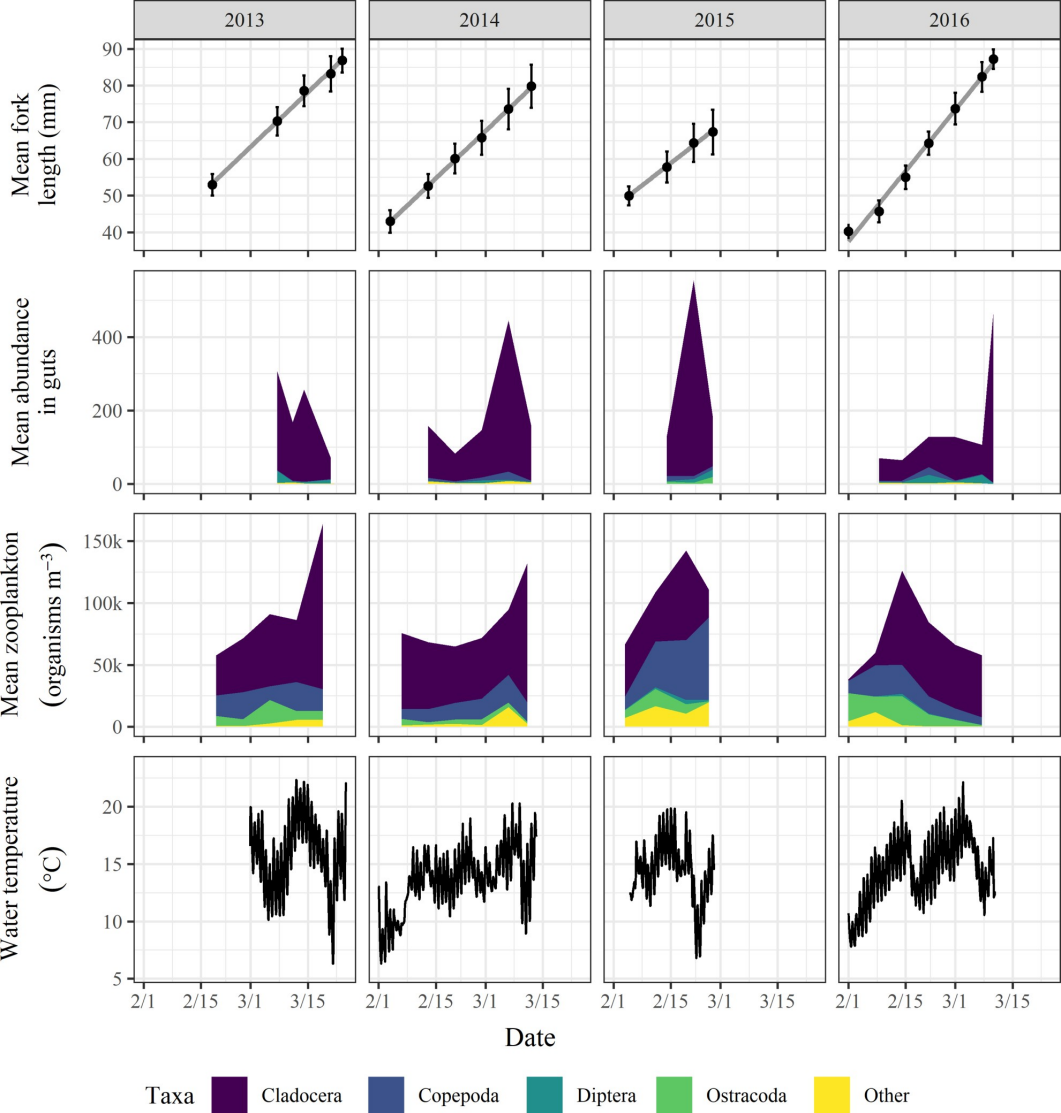
Salmon growth, stomach contents, zooplankton resources, and water temperatures in winter-flooded rice fields. Plot rows from top to bottom: mean fork length (mm) with standard deviation bars and linear regression line representing growth rate (grey), mean prey organism abundance in salmon stomach contents, mean density of in-field zooplankton (organisms m^-3^), and water temperature (°C) in the middle of a representative field (field 4) for each year 2013–2016 in columns displayed from left to right.

Salmon consistently showed a preference for cladoceran zooplankton as this taxon comprised >90% of the stomach contents in all years compared with ambient, in-field cladocera percent composition ranging from 16.4–56.0% (Table 2, Fig 4). Mean prey organism abundance in the stomach contents for each year ranged from 158.3 ± 36.0 SE in 2016 to 278.9 ± 27.6 SE in 2013 indicating that invertebrate food resources were abundant in all years (Fig 4).

### Overall salmon survival and growth

Survival between years was variable, ranging from 7.4% in 2013 to 61.6% in 2016 and increased over the course of the multi-year experiment (Table 3). An increase in survival was observed after 2013 when an undersized culvert in the drainage canal was replaced allowing for much more rapid field draw down in subsequent years.

**Table 3:** Mean percent survival, apparent fork length growth rate and apparent weight growth rate for each year of the experiment.

Year	Mean survival	Fork length growth rate (mm d^-1^)	Weight growth rate (g d^-1^)
**2013**	7.4%	0.96	0.19
**2014**	44.6%	0.99	0.14
**2015**	43.5%	0.81	0.12
**2016**	61.6%	1.28	0.21

Juvenile Chinook Salmon apparent growth rates observed in experimental fields were high in all years ranging from 0.81 mm d^-1^ and 0.12 g d^-1^ in 2015 to 1.28 mm d^-1^ and 0.21 g d^-1^ in 2016 (Table 3).

## Discussion

Rearing of juvenile Chinook Salmon within winter-flooded rice fields shows strong potential for reconciling agricultural floodplain land use with habitat needs of an imperiled and economically important fish. Winter-flooded rice fields demonstrated high production of naturally occurring fish food (zooplankton) leading to high growth rates of salmon reared in these environments. As with past fish conservation studies in altered environments [48–50], our adaptive research approach enabled us to successfully answer experimental questions despite unpredictable winter hydrologic and temperature regimes in the Central Valley.

In our studies, post-harvest field substrate did not have a statistically significant effect on the composition or abundance of zooplankton species, nor on growth rates of rearing juvenile Chinook Salmon. Overall, fish growth across all treatments was extremely fast and much greater than previously documented in the Sacramento River channel environments over the last century [26]. Accordingly, we do not recommend a specific post-harvest straw management practice. Instead, we feel that field preparation should be left to the farmer. However, we encourage future research that explores other approaches for enhancing in-field habitats to decrease predation risk for rearing fish.

There is currently limited means of cost effectively providing avian predation refugia for fish on winter-flooded rice fields. We investigated the potential of in-field trenches to provide depth refuge from avian predation, but direct benefits to survival were found to be insignificant in this study. Fields containing perimeter trenches connecting the inlet and outlet structures did however, show higher rates of volitional emigration of salmon and reduced rates of stranding following draining. We speculate that fish used the trenches as migration corridors when emigrating from the fields. Increased rates of volitional egress would further diversify timing of emigration which has been identified as a key component of population stability via the portfolio effect [51]. Additionally, the trenches buffered water temperatures from the daily maximums observed in the middle of the fields, expedited field drainage, and reduced the number of fish stranded during field draw down.

In floodplain river ecosystems, fishes often respond strongly to hydrological dynamics of ascending and descending flood conditions [52–54]. Juvenile Chinook Salmon in the Central Valley have evolved physiological and behavioral strategies for the use and egress from winter-flooded floodplain habitats [25, 55, 56]. Accordingly, the rate of field drainage and inflow conditions in winter-flooded rice fields may provide important cues for rearing juvenile Chinook Salmon. In our study, extending the drainage period and manipulating inflow conditions in the slow drain treatments had a detrimental effect on survival and the best method was a fast drain where fields were drained in a single day. This was likely the result of increased vulnerability to predation and reduced thermal buffering due to a prolonged period with shallower water depths in the slow drain treatments. Again, these results provide a relatively simple management recommendation for farmers in that simple opening of outlet water control structures with rapid drainage appears to be the best method. We encourage exploration of other drainage methods, and production of other species in winter-flooded rice fields may require different draining practices.

An initial mortality of approximately one third was observed in the first week of the 2016 salmon survival through time experiment. The cause of this initial mortality is unknown and could have resulted from a combination of factors, including a stressful transport and acclimation stress under new physical water conditions. Additionally, because we know of no other experiments that have been able to track and assess post-release mortality of hatchery fish through time, we cannot rule out the possibility that the high rate of initial mortality observed immediately after release into the “wild” is a potentially common phenomenon. Transport is a known stressor on many fishes, including juvenile Chinook Salmon [57, 58]. In our study, fish were captured from hatchery raceways, coded wire tagged, allowed to recover for several days and then placed in a fish hauling tank at high densities (up to 25,000 fish m^-3^) and delivered to the fields in early February. Exposing naïve hatchery salmon to a new environment in the flooded agricultural fields may have increased stress as it necessitated behavioral adaptations of prey switching and predator avoidance as well as rapid acclimation to the new physical water quality parameters. After the high rate of initial mortality, survival stabilized in week two and remained high for the remainder of the experiment. Without accurate assessment and accounting of initial post-release mortality there is potential for fishery managers to be chronically overestimating habitat-specific mortality rates determined by recapture of hatchery fish transported and released into natural habitats. We therefore recommend that future research examine effects on initial post-release mortality of transporting, acclimatizing, and releasing hatchery fish into the wild.

While farmers have incentives to prepare their fields for a new rice crop as early in the spring as possible, fish conservationists may theoretically prefer to keep the rice fields inundated as late as possible to maximize fish growth and survival before release into the river [28, 56]. However, in practice, when weather conditions on the floodplain are good for fish (i.e., wet and cool) in the late winter and early spring, they are generally not conducive to agricultural field preparation. The inverse is also true, as spring conditions become dry and hot and generally suitable for agricultural field preparation, water quality conditions (especially water temperature) rapidly become unsuitable for juvenile Chinook Salmon [59, 60]. Thus, given proper timing and coordination within an adaptive management framework, farmers and fish conservationists can collaborate to promote threatened fisheries without impacting crop yields [26]. We therefore encourage the continued development of adaptive frameworks for the integration of floodplain fish habitat into farm operations that reconcile the needs and timing for both fish and farm operations. Incentive programs for farmers (e.g., through the USDA NRCS or similar programs) may be needed to promote these activities to their fullest potential.

**Fig 5:**
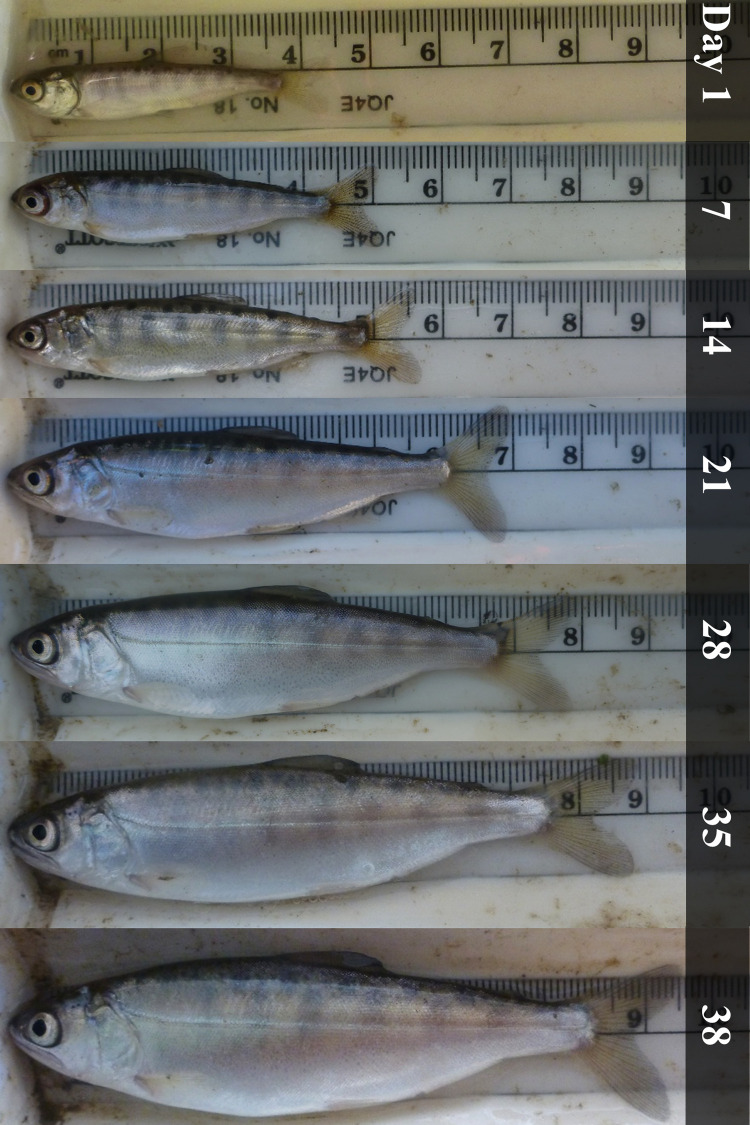
Weekly salmon growth images. Standardized images showing an approximately mean size juvenile Chinook Salmon from each sampling period during the 2016 survival through time experiment. Fish in the images had a unique specimen ID with associated physical size data from field measurement.

Land manager and farmer involvement has generally exceeded expectations in our projects, and we are optimistic about continued stakeholder involvement. Given issues with water scarcity in the Central Valley [61, 62], the dual-use of rice fields for agriculture and rearing juvenile salmon could establish stronger water security for farmers [63]. Additionally, off-season inundation of rice fields promotes rice straw decomposition while approximating the natural long-duration inundation patterns that fuel a productive aquatic food web [24, 64]. When compared to concurrent samples in the adjacent Sacramento River channel habitat, the winter-flooded rice fields had ≥ 150x zooplankton abundance in 2013 [24] and approximately 53x zooplankton abundance in 2016 [29]. Resultantly, the juvenile salmon growth rates we observed in winter-flooded rice fields were 2-5x higher than previously or concurrently observed in the adjacent Sacramento River [27, 29]. By creating high quality habitat on their fields, farmers can help bolster fish populations by rapidly turning small fry into large, healthy smolts during mid-winter when water temperatures are low, river flows are high and when predators are less active, thus improving salmon survival rates during outmigration (Fig 5) [65].

Managed inundation of rice fields in winter and early spring appears to mimic historical Sacramento Valley floodplain processes, re-exposing salmon to an approximated version of the hydrologic selection regime under which they evolved and to which they are adapted. The exceptional productivity and resulting rapid rates of salmon growth documented on the managed agricultural floodplain lead us to conclude that winter inundation of rice fields creates high-quality rearing opportunities for juvenile Chinook Salmon. Although these studies suggest that agricultural landscapes can function as high-quality rearing habitat for juvenile Chinook Salmon, our results should not be interpreted to diminish the conservation need for restoring naturally functioning floodplains where feasible or to suggest that suitable natural (i.e. non-agricultural) habitats are not essential to establishing self-sustaining runs of naturally produced Central Valley Chinook Salmon. Rather, these data demonstrate the potential to reconcile management of agricultural floodplain landscapes with the conservation of wild Chinook Salmon populations through slight modification and reoperation of existing agricultural infrastructure. Managed agricultural floodplains are likely to become another important means for fishery managers to produce ecologically functioning off-channel habits for imperiled native fish, especially during times of low water when remaining natural floodplain habitats do not inundate and are therefore inaccessible to salmon populations confined to leveed stream channels.
